# Hospital Episode Statistics and trends in ophthalmic surgery 1998 – 2004

**DOI:** 10.1186/1471-2415-6-37

**Published:** 2006-12-04

**Authors:** Lucy C Clarke, Scott G Fraser

**Affiliations:** 1 Sunderland Eye Infirmary, Queen Alexandra Road, Sunderland, Tyne and Wear, SR2 9HP, UK

## Abstract

**Background:**

Hospital episode statistics (HES) is a UK national database for the National Health Service (NHS), now available online. The purpose of this study was to observe trends in ophthalmic operations performed during the period from 1998 to 2004, using this data.

**Methods:**

From the 'Main Operation' codes within the 'Free data' section of the HES website we analysed data in regard to 28 specific ophthalmic operations. These represented each sub speciality within ophthalmology.

**Results:**

The figures show a change in the total number and proportions of operations performed for many of the procedures. For example, there was an increase in numbers of orbital decompressions, but a decrease in numbers of glaucoma filtering operations. Changing trends could be seen in different surgical areas such as the change in operations used for corneal grafting and in retinal surgery.

**Conclusion:**

The HES database represents an important, potentially useful source of information. There are imitations in interpretation of and validity of such data related to coding inconsistencies. We suggest the benefit of the data comes from observing trends rather than exact numbers. As other studies using this data have suggested, it is important that clinicians are involved in improving the quality of this data.

## Background

Hospital Episode Statistics (HES) is the national statistical data warehouse for the NHS in England. It includes clinical and administrative information about the care provided to NHS patients who live, or are treated in England. The data is compiled from information exchanged between healthcare providers such as hospital trusts and primary care trusts (PCTs), and the commissioners of that care, primarily the PCTs. This data is submitted via the NHS Wide Clearing Service (NWCS) who forward it to the commissioners, but also copy the information to a database. At fixed times during the year this accumulated data is then set to HES, so whilst the NWCS data is continuously updated, the HES data reflects the fixed period of time of a financial year.

Data is collected by patient administration systems within over three hundred NHS trusts. As well as epidemiological data, there is a large amount of clinical data recorded such as primary diagnosis, main speciality contracted, and health care resource grouping. The Main Operation is identified as the first recorded, and normally the most resource intensive procedure performed during that episode, although no nationally agreed definition exists. The operation codes are taken from the Office of Population Censuses and Surveys tabular list of surgical operations and procedures fourth revision (OPCS4). HES data comprised of over 13 million episodes of treatment provided to patients in the last financial year available 2003/04. The report for the financial year 1998/9 was the first to be made available online, prior to this it was produced as paper based publication only.

The purpose of this study was to observe some trends in ophthalmic operations performed during the period from 1998 to 2004, using the HES data available online [1].

## Methods

There are various categories of data available within the HES database. Within the free data section, the codes for ophthalmic surgery begin with the letter 'C'; the procedures are then listed by operation type. The three digit codes identify broad categories of operations e.g. C18 = correction of ptosis of eyelid. The four digit codes identify subdivisions of that operation according to technique or method, e.g. C18.6 = correction of ptosis of eyelid using aponeurosis technique.

In many cases there are a number of possible ways of coding for the same operation depending on the semantics used in the data entry. For example, a blepharoplasty could feasibly be listed as: excision of redundant skin blepharoplasty (C13.1–13.4), reconstruction of eyelid (C14.8), correction of deformity of eyelid (C15.8), or plastic repair of eyelid (C16.8). For this reason we have limited our analysis of the data to a few operations for each sub-speciality where there is no coding ambiguity, and reflect an interesting trend.

With the exception of the latest available year, information regarding the age of the patients is given as both an average (mean) and non-contiguous age bands 15–59 and 75+. The percentage of paediatric cases of each procedure is therefore not available for all but 2003/4.

## Results

Table 1 shows the total number of each surgical procedure performed for each financial year by the entry code recorded. The last column indicates the gross percentage change over this the time data were available. Over the period 1998 – 2004 the UK population rose by 1 million [2], representing a 1.6% overall increase.

**Table 1:** HES data for various eye operations 1998–2004

		98/99	99/00	00/01	01/02	02/03	03/04	% change over time
Code	Operation							
	**Oculoplastics**							
1	Excision of eye	1025	1000	989	921	1002	924	-9%
1.1	Exenteration	44	50	35	65	47	66	+50%
1.2	Enucleation	583	535	529	475	542	467	-20%
1.3	Evisceration	392	412	422	374	402	382	-3%
								
6.3	Decompression	168	164	203	198	227	230	+37%
								
18	Ptosis total	2457	2710	2662	3170	3307	3780	+54%
18.1	Levator muscle	1126	1239	1352	1373	1433	1616	+43%
18.2	Frontalis muscle	44	47	40	42	40	47	+7%
18.3	Fascia sling	163	120	181	163	136	131	+20%
18.5	Tarsomullerectomy	162	158	155	149	144	163	<1%
18.6	Aponeurosis repair	298	422	418	498	538	576	+93%
18.8	Ptosis other	658	718	734	939	1011	1246	+89%
								
	**Strabismus**							
31–37	Strabismus total	13057	12358	12465	11750	11782	12021	-8%
31	Combined muscle	6612	6385	6495	6039	5937	6309	-5%
32	Muscle recession	3723	3345	3417	3200	3155	2735	-27%
33	Muscle resection	1195	1087	942	966	969	1043	-13%
								
	**Cornea**							
46	Plastic ops cornea	2257	2247	2269	2130	2127	2201	-3%
46.2	Lamellar graft	181	164	205	225	258	288	+59%
46.3	Penetrating graft	1578	1674	1652	1426	1435	1405	-11%
								
	**Glaucoma**							
60	Filtering ops total	9185	6909	5920	5354	5011	4957	-46%
60.1	Trabeculectomy	8622	6415	5421	4751	4282	4206	-51%
								
	**Cataract**							
71	Extracapsular	8596	6483	5228	6119	6892	5385	-37%
73.3	PC capsulotomy	10423	11718	10964	9319	8523	7550	-28%
75.1	Lens insertion	191540	201979	230663	240210	261963	291477	+52%
								
	**Retina**							
54	Bucking for RD	3791	3507	3322	2846	2779	2469	-35%
79.1,2	Vitrectomy	7308	8042	8483	9360	9319	10616	+45%
86.5	Fluorescein angio.	3676	3610	3111	3178	2821	2336	-36%

### Oculoplastics

The number of total excisions of eyes as remained roughly stable. There has been a large increase in the number of orbital decompressions of nearly 40%. Larger still is the percentage increase in ptosis surgery (54%). Most of the subgroups are of fairly consistent proportion, half of which are recorded as being done by levator muscle technique, with the aponeurosis technique representing only 1/6^th^. The group titled 'other' has a large and steadily rising proportion.

### Strabismus

The total number of strabismus operations is slightly down (8%) over the time period. The proportion of combined muscle, recession, or resection operations performed is reasonably stable.

### Cornea

Plastic operations on the cornea (which encompasses lamellar and penetrating grafts, refractive keratoplasty, insertion of corneal prosthesis, and 'others') overall have remained stable. A large percentage increase (59%) in the number of lamellar grafts reflects the small number of this newer operation performed. A far larger number of penetrating grafts are performed, however the numbers are declining a little year on year.

### Glaucoma

Both the total number of filtering operations and the number of trabeculectomies has declined, by 46% and 49% respectively, as described in a previous paper using HES to describe surgical trends [3].

### Cataract

In the period from 1998 – 2004 the total number of prosthesis of lens, (including insertion, removal, revision, and 'other') has increased almost 100,000, an increase of 52%. This is presumably due to the rise in phacoemulsification (although it is not coded as this within HES), given the decline of extracapsular extractions and small numbers of other techniques recorded. A 28% decline in the number of surgical posterior capsulotomies recorded may be due to the use of YAG laser for this procedure, which does not have a surgical code, but may also be in part due to developments in lens technology.

### Retina

An increase in vitrectomy numbers is greater that the decrease in buckling for retinal detachment observed. This may be due to its increased use as relatively novel treatment for other conditions such as macular hole. Fluroscein angiography has also showed a 36% decline, possibly due to increasing use of less invasive technology such as OCT, which is not recorded by HES.

## Discussion

The HES database provides a rich source of information regarding patient care across the country, year by year. The trends identified in this paper represent a fraction of the data available for examination. It is important to acknowledge the limitations of both the data available, and the conclusions that can therefore be drawn from them.

Standardising the many terms used to describe operations would facilitate better comparison of each category by making them less ambiguous. Narrowing or restricting the many terms available such as extirpation, plastic repair, excision, reconstruction, repair, incision and correction of deformity could help to achieve this. The validity of recording a single *'main operation'*, which is not clearly defined, has to be questioned. The use of more complex operations such as phaco-trabeculectomies; and combined surgery, for example ptosis with blepharoplasty, will inevitably lead to an under reporting of the individual components. The use of newer technologies such as laser peripheral iridotomy instead of the surgical equivalent, or novel operations e.g. viscocanulostomy, which are yet to be coded will further erode that validity. Similar limitations to the usefulness of database analysis have been extensively explored in the USA [4,5] where the Medicare claims database has been used to assess the provision of many ophthalmic treatments.

Greater accuracy could also be achieved by direct involvement on the part of the surgeon in the coding procedure. Each practitioner would normally undertake a relatively small number of different operations on a routine basis, so learning and including those specific codes in the operating notes would be possible. It would increase the accuracy of coding and the clinician would also then be responsible for indicating the 'main operation' rather than assignment according resources used.

Surgery is not an exact science, so it is essential to retain some flexibility within the terms used to describe it. Hence the importance of categories 'other – specified/unspecified' for operations that do not accurately match the other codes. However for useful data analysis, this should represent a small minority of the operations performed. In the breakdown of ptosis surgery, one quarter to a third of operations are not classified under the current coding. Other classified techniques show a small variation in numbers performed year to year as a percentage of the total, but unspecified technique is the only subset to show a trend of gradual increase. Superior rectus muscle surgery represents a tiny proportion of the operations performed, in the last year only a single operation recorded countrywide, but continues to retain separate categorisation. In this instance, a re-coding would allow better reflection of current surgical practice. This could be achieved with constant evaluation and clinical input.

The validity of the HES database has been questioned in literature, as acknowledged by the Department of Health. A review article of many of these papers [6] indicates that local variations in coding practice inevitably leads to inaccuracies. As such, it is the trends observed year to year, rather than the absolute numbers represented in the data that are likely to be of greater validity. Given the increasing importance of centralised NHS data in hospital and practitioner performance, they stress the importance of clinician involvement in the process of data collection as well as revision of NHS data terms.

HES has been used extensively by other specialities for a diverse range of data analysis. For example, others have looked at regional variation in joint replacement rates in orthopaedics [7], epidemiological trends in geriatric emergency admissions [8], and mortality rates versus caseload in radical cystectomy [9]. We could only find two published articles utilising HES for analysis of data within ophthalmology. The first article as discussed previously, using HES to show trends in glaucoma surgery [3]. The second providing a comparison of waiting time systems between orthopaedics and ophthalmology within a single hospital [10].

## Conclusion

The use of HES as a free online database has not been referenced widely within the literature related to ophthalmology. This article seeks to show a few simple trends in recent ophthalmic surgical practice, and highlights the potential usefulness of this important UK national resource. However, as stated within the HES documents, care in analysis of the information provided requires an understanding of the quality and the limitations of the data.

## Competing interests

The author(s) declare they have no competing interests.

## Authors' contributions

The study was conceived and executed by LC and SF

LC drafted and completed the manuscript.

SF conceived of, and edited the manuscript.

Both authors read and approved of the final manuscript.

## Pre-publication history

The pre-publication history for this paper can be accessed here:


